# An Active Inference Model of Meter Perception and the Urge to Move to Music

**DOI:** 10.1111/nyas.70129

**Published:** 2025-12-09

**Authors:** Tomas E. Matthews, Peter Vuust, Jonathan Cannon

**Affiliations:** ^1^ Center for Music in the Brain, Department of Clinical Medicine Aarhus University Aarhus Denmark; ^2^ Royal Academy of Music Aarhus Denmark; ^3^ Department of Psychology, Neuroscience & Behaviour McMaster University Hamilton Ontario Canada

**Keywords:** active inference, affordance, Bayesian inference, groove, learning progress, predictive processing, rhythmic music

## Abstract

Why do some rhythms make us want to move and not others? A predictive processing account suggests that prediction errors drive this phenomenon, but this hypothesis remains underspecified. Here, we operationalized this account as a Bayesian model that infers whether a rhythmic sequence is caused by a metered or unmetered template, coupled with an active inference rule in which movement occurs if the sensory feedback from movement would reduce the prediction errors generated by this inference process. Surprisal, as an index of prediction error, was calculated for each rhythm with and without a metronome (a proxy for the feedback from moving along), with delta surprisal as the difference. Surprisal increased linearly as a function of rhythmic complexity, while delta surprisal showed a similar pattern with urge to move ratings shown in previous studies, and this relation was confirmed in an online study. These results suggest that the urge to move to music is driven by the potential to reduce meter‐based prediction errors via the expected feedback from moving along to the beat. This work provides a crucial update to the predictive processing account and highlights a key role of active inference and prediction‐based learning in our musical experiences.

## Graphical abstract

Prominent theories suggest that the urge to move along to rhythmic music is driven by precision‐weighted prediction errors. We operationalized this account as a Bayesian model which outputs surprisal as an index of prediction errors based on posterior probabilities calculated over metered and unmetered priors. Our results suggest that it is the expected reduction in prediction errors due to feedback from on‐beat movements that drives the urge to move.

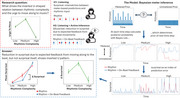

## 1 Introduction

Music is often highly structured in time and thus predictable. This facilitates perceptual processing (e.g., Refs. [1] and [2]) and enables both inter‐ and intra‐individual sensorimotor coordination, which, in turn, promote social bonding [3, 4] and further enhance perception [5, 6], respectively. Musicians intentionally manipulate the predictability of music to modulate the affective and motor responses of listeners [7, 8]. Accordingly, the predictability or complexity of rhythms is a core feature of music, and thus provides an ideal testbed for investigating the link between audio‐motor, perceptual, and motivational processes through a predictive processing lens [9, 10]. In particular, the pleasurable urge to move to music (PLUMM), associated with the larger concept of groove [11, 12, 13, 14, 15, 16], has become a focus of research (see Ref. [17] for a recent review), with a predictive processing account emerging as the most prominent explanation of this phenomenon [18, 19]. This account suggests that precision‐weighted prediction errors (prediction errors weighted by prediction confidence) in relation to the timing of rhythmic onsets are a prominent contributor to PLUMM and drive an inverted U‐shaped relation between rhythmic complexity and PLUMM. That is, moderately complex rhythms allow for relatively confident (precise) temporal predictions but also induce errors, while low complexity rhythms elicit very few prediction errors, and high complexity rhythms elicit very low prediction precision. This inverted U pattern has been shown in a relatively large number of studies using a variety of rhythmic stimuli [16, 20, 21, 22, 23, 24, 25, 26, 27, 28, 29], thus providing tacit support for the predictive processing account. However, several recent studies did not show this inverted U pattern [30, 31, 32, 33], suggesting that the predictive processing account cannot fully explain the rhythmic complexity—urge to move relation. Further, a direct investigation of the process connecting rhythm‐based prediction errors and the urge to synchronize movements to the rhythm is still lacking. Here, we operationalize the predictive processing account of PLUMM in a minimal mathematical model and test whether the inverted U is accounted for by precision‐weighted prediction errors.

The main tenet of the predictive processing framework is that organisms are hardwired to minimize mismatches between sensory input and predictions (prediction errors) produced by generative models of the hidden (i.e., not directly perceptible) causes of this input [34]. These prediction errors are weighted by the precision (confidence) of the antecedent prediction, such that stronger prediction errors lead to greater updates to the generative model (more learning). Prediction errors are calculated in the context of an ongoing process of updating beliefs about the hidden causes in order to minimize prediction error, a process that closely approximates Bayesian inference.^1^

In the predictive processing account of PLUMM, the “hidden cause” of an observed rhythm is a “meter,” a temporal framework that can be thought of as underlying the generation of the rhythm. Within western music scholarship, a meter is a hierarchical pattern of regular time intervals. These intervals are simple multiples of the period of the beat, which is the most salient period that listeners are most likely to move to [35]. Crucially, beat and meter are psychological phenomena, learned via enculturation, that are not necessarily explicit in the sounded rhythm [36, 37]. Beat, in particular, has been framed as a covert motor process in which simulated movements, abstracted away from any specific effector, constitute beat‐based predictions [38]. Therefore, according to these prediction‐based accounts of rhythm perception, a listener will automatically infer beat and meter, which act as a generative model of the rhythmic input. Probabilistic accounts of meter perception are common within the rhythm perception literature [39, 40].

Within PLUMM research, rhythmic complexity is often operationalized in terms of syncopation, which is when an onset on a weakly accented position in the meter precedes a rest on a strongly accented position. In the predictive processing account, syncopations are prediction errors as they go against predictions based on the inferred meter, necessitating an update to this model that is weighted by the precision of the meter‐based prediction [18, 19]. This precision depends on both the position of the event within the meter (i.e., more precise predictions for more strongly accented positions) and the precision of the metric model itself (e.g., certainty that the rhythm is in a 4/4 meter and not a 3/4 meter). Bayesian models of syncopation have been shown to successfully predict subjective judgments of rhythmic complexity [41, 42] and tapping behavior [43].

Extending this to PLUMM leads to the hypothesis that the inverted U‐shaped relation between PLUMM and degree of syncopation exists because rhythms with medium levels of syncopation maximize precision weighted prediction errors, as they engender strong metric models but also have syncopations on strong metric positions [18, 19]. Conversely, highly syncopated rhythms weaken the metric model to the extent that it can produce only low precision predictions, resulting in weakly weighted prediction errors, while minimally syncopated rhythms result in strong metric models but produce very few prediction errors.

However, the specific process by which these precision‐weighted prediction errors engender PLUMM is still unclear. One possibility is that the urge to move results directly from the detection of prediction errors, that is, challenges to the metric model due to syncopations lead directly to an urge to move. Another possibility is that the desire to move reflects “an opportunity to resolve uncertainty and minimize prediction errors in the future” [19, pg. 8] via movement feedback. That is, the sensory and/or proprioceptive input from beat‐aligned movements can reduce surprisal induced by syncopations, and the urge to move may reflect the expected reduction in surprisal if one were to move to the beat. This draws on the predictive processing account of movement, known as active inference, which frames voluntary action as a process of minimizing prediction error by moving the body to fulfill predictions regarding the proprioceptive and sensory outcome of movements (rather than being driven by explicit motor commands [44]).

Here, we operationalize the predictive processing account of PLUMM using a minimal model of Bayesian meter inference. As the rhythm progresses, posterior probabilities are calculated over possible meters at each grid point based on the rhythmic input, that is, onsets or rests. These posteriors then become the priors for the next time point, thus (precision) weighting the prediction based on what came before. This allows for the operationalization of the per‐time‐step precision‐weighted prediction errors as surprisal (the negative log probability of an onset or rest) in a way that accounts for both meter‐based prior probabilities and the preceding context.^2^ To distinguish between precision‐weighted prediction errors and expected minimization of such prediction errors as drivers of PLUMM, we compare patterns of surprisal as a function of syncopation to patterns of “delta surprisal,” that is, the surprisal reduced by adding a quarter note metronome to the rhythmic stream. The metronome serves as a proxy for the feedback from moving or simulating movements along to the beat, so delta surprisal indexes the expected consequences of inferring a beat via overt or covert movements. We then explore the limits of the model specifications within which the relation between PLUMM ratings and rhythmic complexity is accounted for. An online rating study was then implemented to confirm the relation between delta surprisal and wanting to move ratings. We then fit model parameters to these ratings to assess the model specifications that drive this relation.

## 2 Bayesian Meter Inference

We assume that at the beginning of each time step t, the listener entertains a probabilistic prior belief that the rhythm is metered (as opposed to unmetered—uniformly random) which they update to calculate a posterior in light of their observations on that time step. We represent the prior probability with $P_{metered}^{prior}\left(t\right)$, where $1-P_{metered}^{prior}\left(t\right)$ is the agent's prior probability that the rhythm is unmetered. Every simulation begins with a prior probability $P_{metered}^{prior}\left(1\right)$. At every time step t, the belief at the previous time step is used as a prior for a Bayesian calculation that incorporates the observation on that time step into a posterior belief following that observation.

We start by calculating the probability of the observation (obs= onset or rest) on time step t:

(1) $$\begin{array}{ccc} P\left(obs,t\right)=P_{metered}^{prior}\left(t\right) & & \times \;P\left(obs,t|metered\right)+\left[1-P_{metered}^{prior}\left(t\right)\right]\\ & & \times \;P\left(obs,t|unmetered\right). \end{array}$$

We calculate the posterior with Bayes rule as:

(2) $$P_{metered}^{post}\left(t\right)=\frac{P_{metered}^{prior}\left(t\right)\times P\left(obs,t|metered\right)}{P\left(obs,t\right)}.$$

**FIGURE 1:**
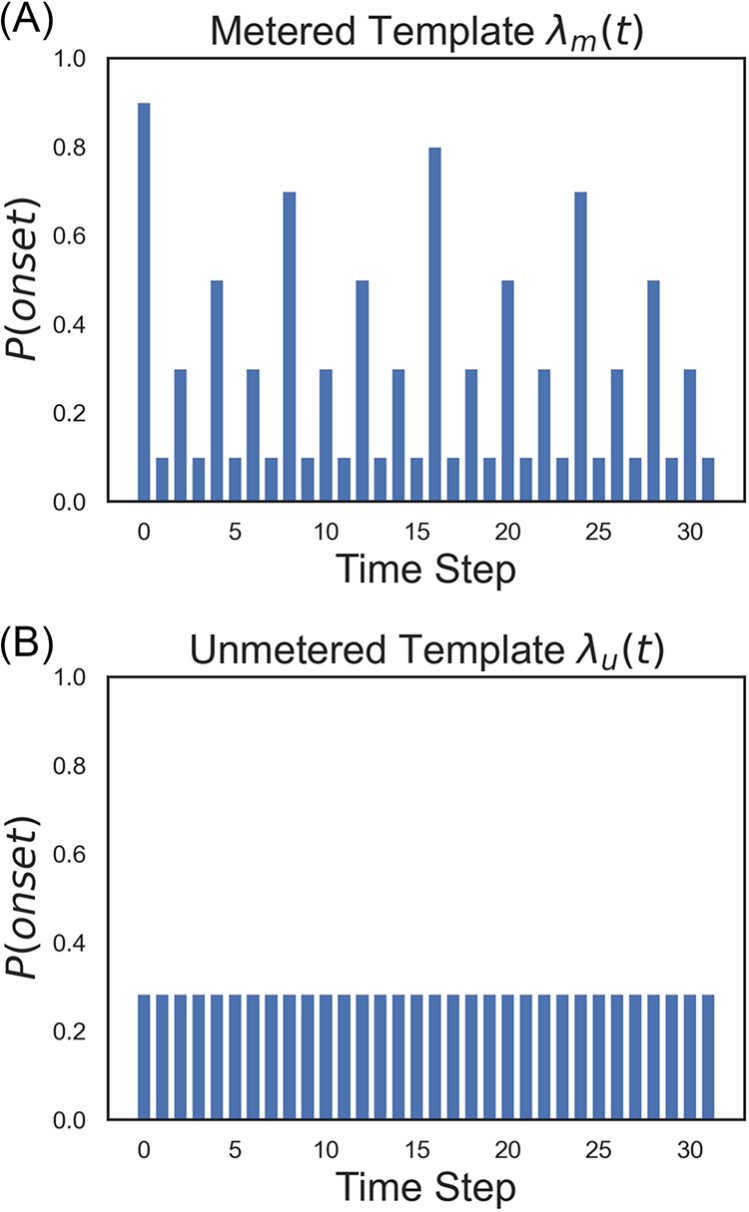
The metered (A) and unmetered (B) templates.

The probabilities P(obs,t|metered) and P(obs,t|unmetered) are determined by two expectation templates (metered and unmetered, Figure 1) that describe the listener's implicit assumptions (i.e., their generative model) about the probability of a sound onset at each time point t. If a rhythm is metered, then an onset occurs at time point t with probability $\lambda_{m}\left(t\right)$, and if it is not metered, then an onset occurs at grid point n with probability $\lambda_{u}\left(t\right)$. If an onset is observed at time point t, then $P\;\left(obs,t|metered\right)=\lambda_{m}\;\left(t\right)$ and $P\;\left(obs,t|unmetered\right)=\lambda_{u}\;\left(t\right)$, and if a rest is observed, then $P\;\left(obs,t|metered\right)=1-\lambda_{m}\;\left(t\right)$ and $P\;\left(obs,t|unmetered\right)=1-\lambda_{u}\;\left(t\right)$.

The metered template $\lambda_{m}\left(t\right)$ consisted of a 32‐step sequence of probabilities based on a 4/4 meter with 32nd note granularity. The probabilities are based on the event weights from Longuet‐Higgins and Lee's [45] model used for calculating a syncopation index. Based on western musical tradition, the weights are assigned hierarchically according to the beat durations present within a 4/4 meter [36], where the longer the duration, the stronger the weight. For example, a whole note, which spans the entire length of a measure, has the largest weight, while a 32nd note only spans 1/32 of a measure and has the smallest weight. Here, we adapt this in probabilistic terms such that each position in a metrical grid is assigned a probability according to its metric weight. As can be seen in Figure 1, the first position has the highest probability, followed by position 16 (half note duration), then the quarter note, eighth, 16th and 32nd note durations. Therefore, according to this model, a listener familiar with a 4/4 meter will have the strongest expectation of a note on the first time step and the lowest expectation on the 32nd note positions (t = 2, 4, 6, etc.).

These theoretical probabilities are given empirical support by the frequency of onsets calculated from a large corpus as well as participants’, particularly musicians, goodness‐of‐fit and discrimination ratings [46]. The corpus used in that study included rhythms with only 16th note durations and longer. Here, the high complexity rhythms included onsets in the 32nd note positions; therefore, we included 32nd note probabilities so that the templates and rhythms match in terms of granularity. Implicit in this choice is the assumption that listeners adjust their expectation templates to match the granularity of rhythms heard within a listening session.

The unmetered expectation template $\lambda_{u}\left(t\right)$ consisted of a 32nd note grid with identical probabilities, determined as the mean probability from the metered template. Therefore, the unmetered template represents an equal level of expectation at all metric positions. Both templates were repeated four times to match the length of the presentation of the rhythms in rating experiments [20, 22, 23, 25].

At the end of each time step t, the posterior from that step was used to calculate a prior for the next time step. In this calculation, we assume that the listener's implicit beliefs (i.e., their generative model of rhythm) allow the rhythm to transition from metered to unmetered or vice versa at the end of any time step with probability $P_{switch}$. Thus, we set

(3) $$\begin{array}{ccc} P_{metered}^{prior}\left(t+1\right) & & =\left(1-P_{metered}^{post}\left(t\right)\right)\times P_{switch}\\ & & +\;P_{metered}^{post}\left(t\right)\times \left(1-P_{switch}\right). \end{array}$$

In this way, the (generative) model captures schematic predictions learned over years of enculturation with western music as well as veridical predictions which adapt based on ongoing input. However, the repetitive nature of these stimuli may attenuate the contribution of veridical predictions compared to more naturalistic listening contexts.

Surprisal S(t) was calculated at each time step t as the negative log probability of an observation (onset or rest), where the probability of an observation P(obs,t) is calculated by marginalizing across both possible templates (Equation 1). Therefore, surprisal quantifies how surprising the observation is given the listener's probabilistic beliefs about the meter (the generative model) and the template‐based predictions those beliefs imply about the present observation given the preceding context, thus providing an index of precision‐weighted prediction error.

(4) $$S\;\left(t\right)=-\log\left(P\left(obs,t\right)\right)$$

Surprisal was averaged over t to calculate a total mean surprisal value for each presented rhythm.

To test whether the urge to move is driven by the detection of precision‐weighted prediction errors (surprisal) or the expected prediction error minimization, we added a quarter note metronome to the rhythmic pattern as a proxy for the sensory or proprioceptive feedback that a listener receives when moving along to the beat (see Stimuli). We measured the degree to which mean surprisal is reduced by the metronome by subtracting the mean surprisal from each rhythm with the metronome from that without the metronome, calling the difference “Δ Surprisal.” This delta surprisal measure indexes the degree to which precision‐weighted prediction error is expected to be minimized by moving along to the quarter‐note beat of a given rhythm.

Note that our model differs from previous Bayesian models of rhythm perception and syncopation. Specifically, the current model accounts for precision weighting by updating posteriors on a per‐time‐step basis. Conversely, Fram and Berger's [41] model does not include an updating mechanism, and Senn's [42] model projects posteriors onto the next corresponding position in the meter (e.g., from the strong first beat of the first measure to the strong first beat of the second measure), which is framed as pattern learning. Therefore, neither model explicitly accounts for precision weighting. Van der Weij and colleagues [47] provide another Bayesian model of meter perception built on the Information Dynamics of Music (IDyOM) model [48, 49], but focus on the role of enculturation. Meanwhile, Cannon's PIPPET [43] model focuses on beat and rhythm perception and includes phase and tempo inference in addition to template inference (similar to our meter inference), and sets the inference process in continuous time. Conversely, the current model assumes that the metrical phase is known and does meter inference at discrete time steps determined by the fastest metrical level represented in the rhythms. Therefore, previous models either do not explicitly account for precision weighting or were designed to serve different goals than the current model, which focuses on the active inference processes thought to drive PLUMM.

$P_{switch}$ determines the probability of transitioning between the metered and unmetered template at each time step (according to the listener's generative model) and thus will have a strong influence on the calculation of surprisal and delta surprisal—specifically, $P_{switch}$ determines the rate at which $P_{metered}^{prior}$ drifts toward 0.5 (in addition to its ongoing adjustment to new rhythmic information). Thus, a listener with low $P_{switch}$ is able to maintain a persistent sense of meteredness (or nonmeteredness) for longer without strong confirmatory evidence, while a listener with high $P_{switch}$ loses their sense of meteredness without strong confirmatory evidence. The pattern of probabilities within the metered template, specifically the differentiation between strong and weak metric positions (referred to here as metrical contrast C), will also affect the surprisal and delta surprisal values. In effect, this parameter modulates the difference between high and low probability positions (as visualized in Figure 1A) with lower values of C reflecting smaller differences between these positions and thus a “flatter” metered template. Therefore, this parameter can be thought of as the degree to which a listener unconsciously distinguishes between probabilistically strong and weak metric positions. We ran the model with four values of each of these parameters, covering a large range within which the pattern seems to stabilize ($P_{switch}$ =0.01,0.1,0.2,0.3;C=0.1,0.33,0.66,1.00). For $P_{switch}$, higher values reflect a higher probability of transitioning from one template to the other. Metrical contrast was manipulated as a scaling factor between zero and one that alters the relative differences between probabilities while keeping the mean constant:

(5)

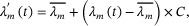

where $\bar{\lambda_{m}}\;$ corresponds to the mean probability across all time steps. C =1.00 reflects the original metered template based on Longuet‐Higgins and Lee [45]. As C decreases, the metered template becomes flatter or less differentiated, with smaller differences in probability between strong and weak metric positions. The main results (Figures 3A and 4) are produced with *C* = 1.00 and *P*_*switch*_ = 0.01, reflecting a highly differentiated metered template with a low probability of shifting to the unmetered template (high persistence of metricality).

### 2.1 Stimuli

**FIGURE 2:**
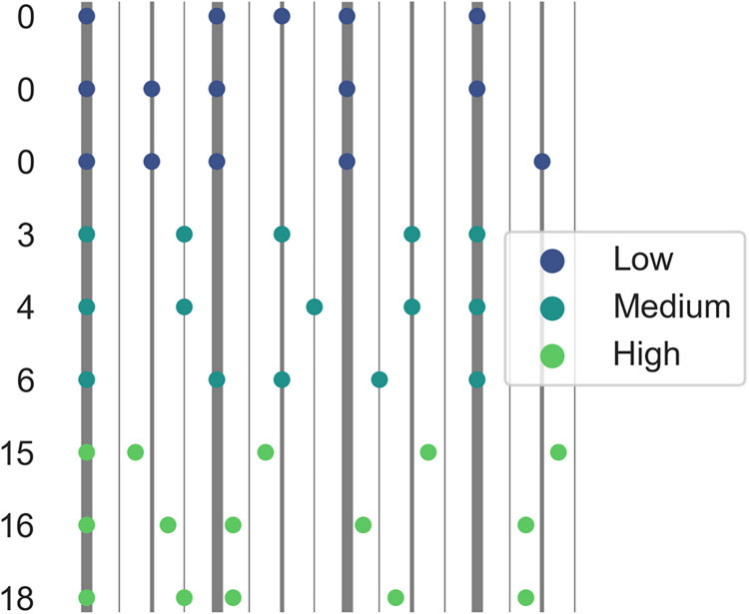
Rhythmic patterns with summed syncopation values on the y‐axis. Thick vertical lines delineate the quarter note beat and thus where the metronome onsets fall. The colors indicate the level of rhythmic complexity. Each stimulus consisted of four repeats of each pattern.

Nine rhythmic patterns, each with five onsets along with an isochronous eighth note hi‐hat, were chosen as input to the model (Figure 2). These patterns were initially created for a rating study testing the effect of rhythmic (and harmonic) complexity on PLUMM [25]. This stimulus set, or subsets thereof, have been used in more recent studies replicating the inverted U‐shaped relation between PLUMM and rhythmic complexity in young healthy participants [20, 22, 23, 25]. Rhythmic complexity is operationalized as the degree of syncopation, calculated according to the approach of Longuet‐Higgins and Lee [45] delineated in Fitch and Rosenfeld [36] (see also Ref. [23] for examples of applying this method to examples of the stimuli used here). In this method, beginning with a metric template, a syncopation occurs when a note precedes a rest, where the rest has a stronger metric weight than the preceding note. The difference between the two metric weights involved in the syncopation determines the strength of the syncopation. Syncopation strengths are then summed within a rhythm as an index of the complexity of that rhythm. Here, as in previous studies using these stimuli, we consider rests on eighth note positions or higher when calculating syncopation values. That is, an onset on a 32nd note position preceding a rest on a 16th note position does not contribute to the syncopation value.

Medium complexity rhythms consisted of clave‐type patterns (e.g., the son and rhumba claves) that are common across genres, particularly within dance‐oriented music. For the low complexity rhythms, the syncopations were removed by shifting off‐beat onsets onto the quarter or eighth note beat positions. For the high complexity rhythms, all onsets except for the first were shifted to an even weaker metric position (mostly 32nd note positions). All rhythmic patterns included an onset on the first beat.

In the metronome condition, we added onsets at the quarter note position (shown as dark bars in Figure 2) to all stimuli. As input to the model is a single stream, quarter note positions that already have an onset are unaffected.

**FIGURE 3:**
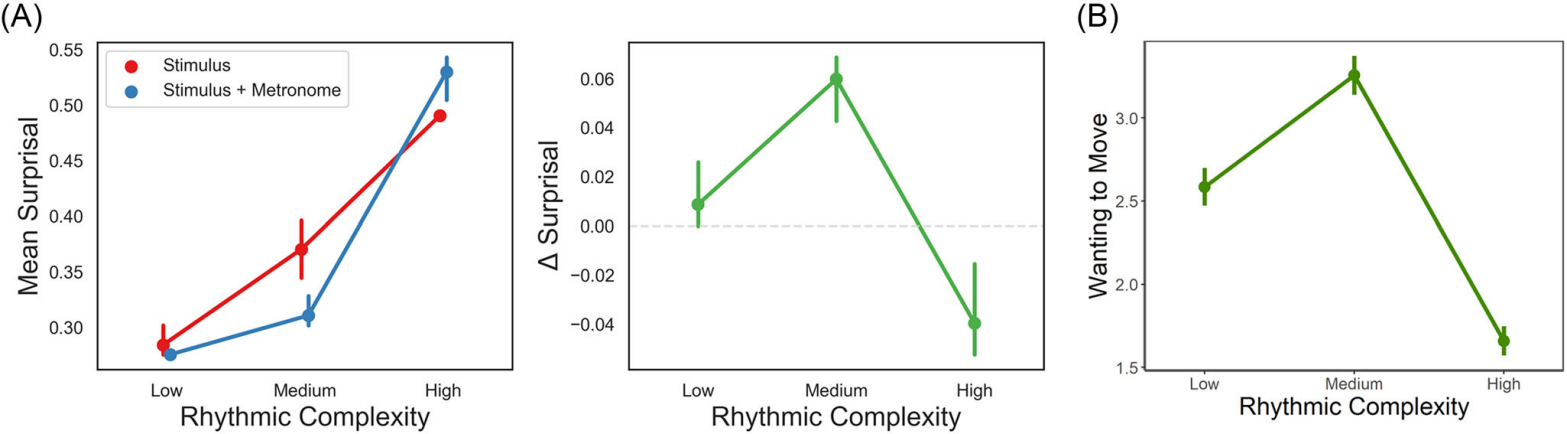
(A) The left panel shows surprisal as a function of rhythmic complexity for rhythms with and without a quarter note metronome, while the right panel shows the subtraction of the two (Δ surprisal). Both panels, $P_{switch}$ =0.01;C=1.00. (B) Ratings of the same rhythmic patterns from Ref. [25] from 201 healthy participants (mean age 34.72, SD = 13.42, 96 identifying as female). Ratings are averaged within each level of rhythmic complexity, within participant. Error bars in all plots reflect 95% confidence intervals.

To compare the pattern of mean surprisal and delta surprisal values with the pattern of urge to move ratings, we plot ratings from a previous online study (Figure 3, Ref. [25]). In that study, 201 healthy participants (mean age 34.72, SD = 13.42, 96 identifying as female) listened to and rated 54 auditory stimuli based on the degree to which they elicited an urge to move along and pleasure (both on a 1–5 Likert scale; only urge to move ratings shown here). The stimuli consisted of the same nine rhythmic patterns as those used here (repeated four times, lasting 10 s), delineated by piano chords along with an eighth note isochronous hi‐hat. The chords also varied along three levels of harmonic complexity; therefore, the 54 stimuli used in that study were a subset of all 81 possible combinations of nine rhythmic patterns and nine chords (three per complexity level). Therefore, participants rated each rhythmic pattern six times, each with a different piano chord. For Figure 3B, the ratings were averaged to generate one mean rating value per level of rhythmic complexity, per participant.

### 2.2 Results

#### 2.2.1 Mean Surprisal and Delta Surprisal

As Figure 3A (left panel) shows, mean surprisal increased linearly as a function of rhythmic complexity for the stimuli without the metronome. Adding the metronome led to a decrease in mean surprisal for medium complexity rhythms and an increase for high complexity rhythms. Accordingly, delta surprisal, the result of subtracting the stimuli + metronome surprisal from the stimuli only surprisal, showed an inverted U‐shaped relation with rhythmic complexity (Figure 3A, right panel). This matches well the pattern seen in ratings of the same stimuli from healthy participants (Figure 3B), with medium complexity rhythms showing the highest delta surprisal and a relatively small difference between low and medium, and a larger difference between medium and high.

#### 2.2.2 Dynamics of Posteriors and Surprisal

**FIGURE 4:**
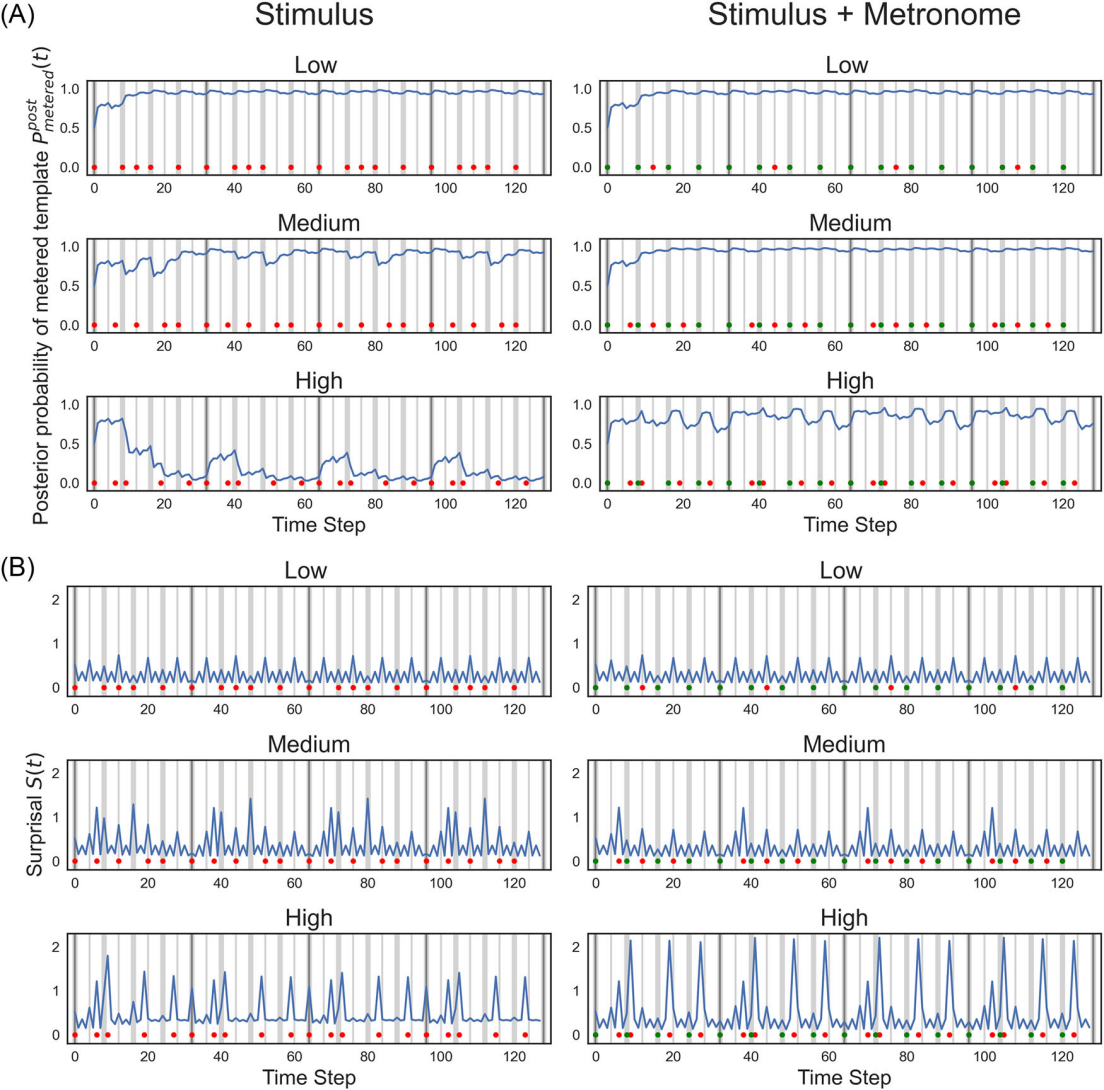
(A) Posterior probabilities $P_{metered}^{post}\left(t\right)$ over 128 time steps for three example rhythms from each level of complexity with (left panel) and without (right panel) the metronome. Higher values indicate the metric model and low values indicate the metered template. (B) Surprisal values S(t) over 128 time steps for the same three rhythms. Red dots, rhythm onsets; Green dots, metronome onsets. Dark gray vertical lines, first beat of pattern; lighter gray vertical lines, quarter note (thick) and eighth note (thin) positions.

Figure 4A shows the dynamics of posterior probabilities over time, with and without the metronome for exemplars of low, medium, and high complexity rhythms. Figure 4B shows the corresponding surprisal dynamics. The posteriors of the low complexity rhythm without a metronome quickly move toward the metered template and stay there. There are small dips in the posteriors and corresponding small spikes in the surprisal when onsets or gaps fall on the slightly weaker eighth note positions. This pattern is not affected by the presence of the metronome as all metronome onsets coincide with rhythm onsets.

The posterior probabilities for the medium complexity rhythm without a metronome show more variability but generally stay close to the metered template. There are drops in the posteriors and corresponding spikes in surprisal on both components of the syncopations, that is, when notes occur on relatively weak metric positions and rests occur on relatively strong positions. Adding the metronome removes the gaps on strong beats, thus removing the dips in the posterior and the spikes in the surprisal; however, the spikes for off‐beat onsets remain.

For the high complexity rhythm without a metronome, the posterior is pulled toward the unmetered template. Once this template is strongly favored, surprisal is tempered by a lack of precise event timing predictions—nothing is especially surprising. When the metronome is added, the posterior is pulled back toward the metered template, leading to surprisal spikes at highly syncopated events and higher mean surprisal overall.

#### 2.2.3 Effects of $P_{switch}$ and C on Delta Surprisal

**FIGURE 5:**
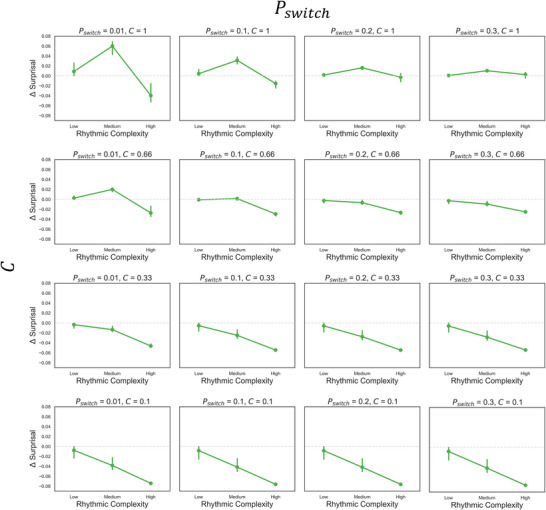
Delta surprisal plots for four levels of $P_{switch}$ (increasing across columns) and metrical contrast C (decreasing across rows).

Figure 5 shows the effects of the switch and contrast parameters on delta suprisal. As $P_{switch}$ increases, the pattern of delta surprisal values flattens out. Decreasing metrical contrast C also leads to a flattened, but also more negative linear pattern of delta surprisal values. This suggests that both the probability of transitioning between templates over time and the differences between strong and weak probabilities strongly influence the shape of the inverted U‐shaped relation between the urge to move and rhythmic complexity. See Figure S1 for the corresponding mean surprisal values with and without the metronome.

## 3 Rating Study

We conducted an online rating study to test the degree to which delta surprisal values predict wanting to move ratings.

### 3.1 Materials and Methods

The study was generated and hosted using the SoSci survey (www.soscisurvey.de) platform. Participants were recruited and compensated via Prolific (ww.prolific.co). The study lasted approximately 20 min. Participants were compensated approximately £3 according to Prolific's recommended rate of £9/h.

### 3.2 Participants

To participate in the study, participants needed to be fluent in English, be between the ages of 18–60, have no hearing difficulties, nor suffer from any neurological or psychological disorders. Participants could only participate in the study once. There were no restrictions in terms of location. Musical training information was collected via the Goldsmiths Musical Sophistication Index, musical training subscale (GMSI) [50].

Two hundred and four participants participated in the study. Data from 21 participants were excluded for failing at least one of the attention checks (details below). Data from an additional five participants were excluded for showing near‐zero variability in their ratings (*n* = 3) or for rating all stimuli as either 100 or 1 (*n* = 2). Therefore, data from 178 participants were included in the analysis. These participants had a mean age of 34.9 (range: 18–60) and had a relatively large range of musical training (mean sum GMSI: 18.54, range: 7–47). Eighty participants identified as female, and one preferred not to say. Participants resided primarily in Africa (*n* = 69), the United Kingdom (*n* = 50), and Europe (*n* = 40). In addition, there were participants residing in India (*n* = 2), Australia (*n* = 1), New Zealand (*n* = 1), Japan (*n* = 1), and Israel (*n* = 1).

### 3.3 Stimuli

Two sets of 30 stimuli were algorithmically generated; one set (Set 1) generated according to a uniform range of delta surprisal values (between −0.047 and 0.093) and one set (Set 2) generated according to a range of syncopation values (between 0 and 18), matching the syncopation values of the original set of nine stimuli used in the above simulation. In this way, the ratings−delta surprisal relation was not biased by how the rhythms were generated.

Both sets were designed to match the original set of nine patterns used in the simulations above. They all included four repeats of five‐onset patterns, had an onset on the first beat position, included an isochronous eighth note hi‐hat, were presented at 96 BPM, and lasted 10 s. The onsets consisted of piano chords in D major with six notes spanning four octaves (D2 to #D5) with a medium level of harmonic complexity (four‐note chords with extensions; see Ref. [25] for details). Within each set of 30 patterns, one of three medium complexity chords were used for each randomly selected set of 10 rhythms.

The stimuli were generated using custom functions based on a previously created Python package for generating rhythmic patterns (https://github.com/OleAd/GrooveGenDist). The patterns were generated based on a list of target values (with a buffer of 0.01 for delta surprisal and 1 for syncopation index) and a set of constraints (five onsets, always a note on the first beat, and no consecutive 32nd note onsets). To facilitate the generation of both simple and complex rhythms within the above constraints, onsets were placed according to a probabilistic template consisting of a weighted combination of the metered and null templates. The weight determining the relative contribution of each template was randomly sampled from a uniform distribution between 0 and 1.

### 3.4 Procedure

Participants listened to the rhythmic patterns and rated the degree to which the rhythm made them want to move using a slider on a 0–100 scale with the corresponding labels “Not at all” and “Very much.” Participants used a desktop or laptop computer and were asked to sit in a quiet room and use the best headphones they had access to. A volume check allowed participants to listen to a rhythmic pattern similar to those used in the study and adjust the volume to a comfortable level. Two auditory attention checks at random points during the study consisted of a voice instructing participants to place the slider on one of two extreme sides of the rating scale. Participants were informed of the attention checks in advance and that if they failed these checks that their data would be unusable.

### 3.5 Data Analysis

Ratings were analyzed using linear mixed effects models with the lme4 package [51] in the R environment. Scatterplots indicated a monotonic relation between wanting to move ratings and both delta surprisal and syncopation values. Therefore, ratings, z‐scored within participant, were analyzed as a linear function of these predictors. First, the relations between ratings and delta surprisal and ratings and syncopation were investigated in two separate models, including GMSI, age, and the rhythms set as fixed effects. All possible interactions were also included. The random structure for both models consisted of per‐participant random slopes for delta surprisal/syncopation, with per‐participant random intercepts suppressed given the within‐participant normalization (i.e., all intercepts are zero). ANOVAs were used to test the significance of interactions and main effects. Reduced models with the same random structure were used to further assess interactions and main effects.

In a second analysis, hierarchical regression was used to test whether delta surprisal accounted for variance in ratings over and above that accounted for by syncopation index. Here, the other fixed effects were excluded, and the random structure was the same as above.

In all cases, *p*‐values were calculated with the lmerTest package [52], with denominator degrees of freedom estimated using the Kenward−Roger method. Diagnostic plots were used to check for violations of assumptions, with no violations detected.

### 3.6 Parameter Fitting

To assess the dependence of the simulation results on the chosen parameter values, we applied a parameter fitting procedure to the rating data using the SPSQL algorithm within the *scipy.optimize* module in Python. Two additional parameters were fit along with $P_{switch}$ and C that scaled delta surprisal to match the raw ratings. These parameters reflect the slope and intercept in the equation of a line. For each participant, the algorithm found the four parameter values that minimized the root mean squared error between the delta surprisal values and the ratings. We fit the model five times per participant, with randomly varying starting points on each iteration, and selected the best‐fitting parameter specification from those five iterations. The resulting parameter values were then used to generate per‐participant predicted ratings for the nine rhythms used in the original simulation.

### 3.7 Results

#### 3.7.1 Ratings

**FIGURE 6:**
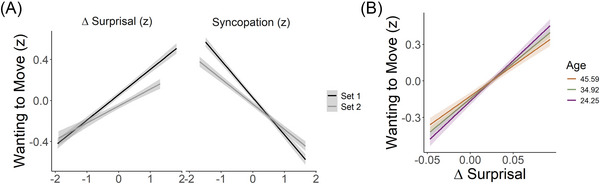
Results of the online rating study. (A) Relation between wanting to move ratings, delta surprisal, and syncopation. Set 1, rhythms generated according to a range of delta surprisal values; Set 2, rhythms generated according to a range of syncopation values. (B) Relation between wanting to move ratings and delta surprisal as a function of age. The three lines reflect mean age (34.92) and mean age ± 1 standard deviation (45.59, 24.25).

An ANOVA showed a significant main effect of delta surprisal (*F*(1, 474.9) = 24.93, *p* < 0.001) and a significant interaction between delta surprisal and age (*F*(1, 474.9) = 3.98, *p* = 0.046). Follow‐up analysis showed a significant positive relation between delta surprisal and wanting to move ratings (b = 8.55, *t*(371.9) = 9.82, *p* < 0.001) and that this decreased with age (b = −0.077, *t*(371.9) = 3.25, *p* < 0.01). As can be seen in Figure 6, delta surprisal showed a strong positive relation with wanting to move ratings for both sets of rhythms. This was supported by the fact that the interaction between delta surprisal and set was not significant (*F*(1, 10490) = 0.0028, *p* = 0.96). No other main effects or interactions were significant.

The ANOVA on the syncopation model showed a significant main effect of syncopation (*F*(1, 2304.9) = 41.3, *p* < 0.001) driven by a strong negative relation with wanting to move ratings (b = −0.056, *t*(1713) = −32.81, *p* < 0.001). No other main effects or interactions were significant.

The hierarchical regression showed that delta surprisal improved model fit when added to a model with syncopation index (χ^2^ = 28.82, *p* < 0.001), indicating that delta surprisal accounts for variance in ratings over and above that accounted for by syncopation.

#### 3.7.2 Parameter Fitting

**FIGURE 7:**
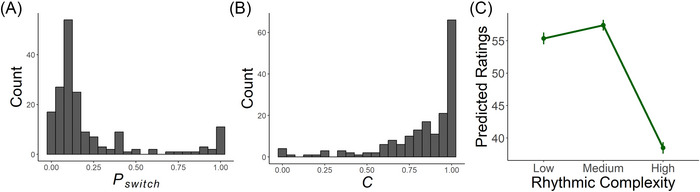
Results of parameter fitting. (A) and (B) show the distribution of $P_{switch}$ and C values from the per‐participant parameter fitting. (C) shows the delta surprisal values for the original set of nine rhythms calculated from the fit parameters.

The results of the parameter fitting are shown in Figure 7. Median $P_{switch}$ (0.107) and C (0.91) values are relatively close to those used in the simulation; however, the histograms (Figure 7A,B) show substantial between‐participant variability. Per‐participant predicted ratings of the original set of nine rhythms show an inverted U‐shaped relation with rhythmic complexity. However, the ratings of the low complexity rhythms are significantly higher. This might be due to the stimuli set used in the online study having fewer low complexity (i.e., zero syncopation) rhythms. Alternatively, this may be due to differences in the sample of participants (e.g., fewer highly trained musicians).

## 4 Discussion

Predictive processes have long been theorized to drive aesthetic, affective, and physical responses to music [7, 53]. Recent theories have emphasized the role of rhythm‐based predictions on the human propensity to move to music [18, 19]. Here, we formalized a predictive processing account of PLUMM in a simple mathematical model that employs per‐time‐step Bayesian inference over metered and unmetered probability templates, outputting surprisal as an index of precision‐weighted prediction error. While surprisal showed a positive linear relation with rhythmic complexity, delta surprisal, as a measure of how much prediction error can be reduced through on‐beat movement, showed an inverted U‐shaped relation; medium complexity rhythms elicited the highest delta surprisal, while low complexity and high complexity rhythms elicited intermediate and lower levels of delta surprisal. This pattern aligns remarkably well with the pattern of urge to move ratings from young healthy participants from an online study using the same stimuli (Figure 3B) [25], a pattern which has been replicated in other studies using the same stimuli [20, 22, 23].

A follow‐up rating study using algorithmically generated rhythms confirmed that delta surprisal positively relates to wanting to move, regardless of whether the rhythms were generated based on a range of delta surprisal or syncopation values. Further, delta surprisal accounted for variance in the ratings over and above that accounted for by syncopation, suggesting that it is not simply an inversion of the syncopation effect. Fitting the $P_{switch}$ and C parameters on this data showed a majority of values near those used in the simulation. Further, delta surprisal values estimated based on these per‐participant parameter values showed an inverted U‐shaped pattern, albeit with somewhat higher ratings for the low complexity rhythms compared to previous results. This suggests that most participants had strongly differentiated metered templates (high values of C), with relatively strong persistence of metricality (low values of $P_{switch}$).

Together, these results provide key support for a predictive processing account of PLUMM while adding an important nuance: that the inverted U is not driven by the detection of the precision‐weighted prediction errors, but by the opportunity to reduce these prediction errors via self‐generated input, for example, the feedback from movements that fill in gaps created by on‐beat syncopations. Accordingly, these results suggest an important role of active inference in PLUMM and align with theories emphasizing the role of learning via prediction error reduction in determining the stimuli that humans choose to engage with.

Figure 3A (right panel) shows that delta surprisal takes on its characteristic inverted U shape because the addition of a metronome has little impact for simple rhythms, decreases surprisal (prediction error) for medium complexity rhythms, and increases surprisal (prediction error) for high complexity rhythms. Some of the prediction errors produced by medium complexity rhythms occur when rests fall on strong beat positions; the sensory or proprioceptive feedback from moving to the beat fills in these gaps, reducing prediction error. Much of the prediction error produced by high complexity rhythms occurs when events fall on metrically weak (i.e., 32nd note) positions; this prediction error is reduced when the listener infers that there is no meter, but in the presence of beat‐aligned movement feedback, they continue to infer that there is a meter, resulting in strong prediction errors.

Figure 3A (left panel) shows that mean surprisal takes on a monotonic increasing shape for rhythms with or without feedback, not an inverted U. This phenomenon is robust to parameter choices (see Figure S1) and seems to be an inherent feature of this type of model. This goes against the predictive processing accounts of PLUMM [18, 19, 54], which hypothesized that the strong syncopations in high complexity rhythms would undermine metric certainty and, in turn, the precision of predictions, resulting in very weak prediction errors. Instead, as we have discussed above, the current results suggest that it is not the strength or precision of the prediction errors per se that drive PLUMM but whether the prediction errors are reducible via expected feedback of on‐beat movements. This aligns with work showing that groove ratings are driven more by the locations of syncopations and the rhythmic patterns they generate, rather than the overall syncopation value [33]. The current results suggest that it is the patterns that are resolvable via movement that will maximize an urge to move.

Although mean surprisal still showed a monotonic increasing pattern, increasing the switch and contrast parameters flattened this pattern and altered the relation between mean surprisal values with and without the metronome (Figure S1), thus altering the pattern of delta surprisal values (Figure 5). As the probability of transitioning between templates increases and the metered templates become less differentiated, the pattern of delta surprisal values flattens out. Therefore, the model generates the characteristic inverted U‐shaped pattern within parameter regimes where there is both high persistence of metricality and strong differentiation between onsets occurring on strong versus weak metric positions. Recent work using these same stimuli has shown that both healthy aging and Parkinson's disease flatten the pattern of PLUMM ratings as a function of rhythmic complexity [20], and that musical training leads to a more peaked pattern [26]. The online rating study revealed a significant but relatively weak interaction between delta surprisal and age, showing a similar flattening effect as in the above studies. However, there was no interaction between delta surprisal and musical training, nor interactions between syncopation and age or musical training. Unlike the aforementioned studies, we did not target highly trained musicians or older populations (or those with Parkinson's), likely reducing the possibility of detecting the influence of these factors. Therefore, by simulating urge to move ratings as delta surprisal within targeted populations, and applying the parameter fitting approach used here, future work will assess whether the switch and/or contrast parameters can account for the effects of healthy aging, Parkinson's, and musical training.

The online rating results did not show an inverted U‐shaped relation between ratings and syncopation. We used algorithmically generated rhythms in order to validate the relation between delta surprisal and wanting to move ratings, and not the inverted U relation with syncopation. However, this result highlights the dependence of the inverted U on the specifics of the stimulus set and that the effect of syncopation is likely proportionate to the number of onsets in the pattern. For relatively sparse patterns, as in the five‐onset patterns used here, the urge to move depends on the presence of only one or two strong syncopations. Therefore, the inverted U depends on the inclusion of multiple very simple (i.e., zero syncopation) rhythms, whereas there is only one algorithmically generated rhythm with zero syncopation in the set used in the online study. Recent work using drum rhythms showed that the number of, and interaction between, polyphonic layers strongly influences PLUMM ratings [27]. Therefore, future work should test whether delta surprisal can account for the urge to move in more naturalistic and complex rhythms, including the role of interacting rhythmic layers.

The main results shown here are driven, at least partially, by the model assumption that a listener can maintain the representation of a 4/4 meter with a specific phase even in the face of highly syncopated rhythms. Without this assumption, we would need to include listener inference about the phase of alignment of the meter with the stimulus, as in Ref. [43]. However, this assumption is reasonable given that in previous rating studies [20, 22, 23, 25, 55], these rhythmic patterns are presented along with an isochronous eighth note hi‐hat, which provides a phase‐specific metrical context and thus prevents a listener from shifting the phase of the inferred meter to better align with the onsets [36]. Therefore, the listener is able to generate expectations regarding feedback from on‐beat movements even in the context of high complexity rhythms. Note also that in a majority of music listening situations, including PLUMM rating experiments, there is an overlying expectation that the auditory input is the product of some underlying structure derived from the intentions of the composer or performer(s), that is, the rhythms will be metered. However, this does not help in terms of reducing prediction errors, and in fact increases them as expected movement feedback reinforces inference of the metered template, making the very weakly metric notes even more surprising. Future work should directly address this assumption by testing participants’ ability to move to more complex rhythms or by precluding this assumption altogether by including tempo and phase inference in the model, as in PIPPET [43, 56, 57].

Another assumption of this model is that listeners will be inclined to make basic, metronomic movements along with a quarter note beat, and that they will experience their feedback from this movement as directly augmenting the heard rhythm. The idea that motor feedback augments a perceived rhythm receives support from theoretical and empirical considerations in Ref. [58]. That listeners will be inclined to make metronomic movements with the beat is, in a sense, baked into the definition of “beat”: in general, the underlying beat of a rhythm is defined not only by its perceptual salience, but also represents the metric level that humans consistently embody [35]. Indeed, PLUMM is generally framed in terms of an automatic urge for simple, on‐beat movements (e.g., bobbing one's head or tapping one's foot). When intentionally dancing, humans do synchronize to faster metric levels; however, these tend to be movements of the extremities that are anchored to the beat, which is embodied by core body regions (e.g., torso, hips) [59, 60, 61]. Recent work suggests that the strong interdependence between rhythmic auditory perception and motor production is rate‐specific, possibly due to mutually adaptive constraints. For example, the beat frequency (tempo) of most music (∼2 Hz) tends to align with the frequency of natural body movements [62, 63], and synchronized movements in this frequency range maximally enhance perceptual acuity [6].

On a theoretical level, our conflation of motor feedback with external sound in a process of active inference is in keeping with action‐oriented accounts of beat perception. The Action Simulation for Auditory Prediction (ASAP) hypothesis suggests that beat‐based sound predictions are mobilized by the motor cortical‐basal ganglia mechanisms that also plan and execute movements and send efference copies to sensory areas to predict the movement's sensory feedback [38, 64]. The ASAP hypothesis is supported by neuroimaging studies that have consistently shown engagement of motor cortical‐basal ganglia networks during beat‐based rhythm perception, even without movement [65, 66, 67, 68, 69]. Under this hypothesis, the perception of a beat is akin to simulating beat‐entrained movement and its consequences, making the calculation of expected surprisal by movement straightforward. The idea that motor simulation supports calculation of expected surprisal (if the listener was moving) and thus drives the urge to move is supported by the observation that groove is linked to excitability in the motor cortex, particularly in musicians, and when this excitability is driven by on‐beat stimulation [70]. Further, urge to move ratings have been linked to activation within motor cortical‐basal ganglia networks [24, 55].

Our model makes the additional simplifying, and perhaps unrealistic, assumption that the expected motor feedback corresponding with the inferred beat is represented within the same input stream as the external rhythm. However, we note that the periodic vestibular input produced by characteristic spontaneous beat‐induced movements, including head‐bobbing, is highly effective at inducing a beat percept [71, 72], and may, therefore, be reasonable to include alongside the auditory input as part of the metrically relevant input stream. More generally, beat‐based motor activation seems to occur across multiple sensory modalities [73], and behavioral evidence shows auditory‐tactile integration during meter inference [74]. Future iterations of this model will segment the expected motor feedback (the metronome) and auditory input into separate streams. Comparing these with the current model will provide further insight into how the beat is represented in the brain.

This work also aligns with a recent account suggesting that PLUMM is at least in part driven by the intrinsic motivation for learning via prediction error reduction [75]. This is based on the Learning Progress hypothesis, which suggests that the rate of prediction error minimization over time is itself a reward signal [76, 77]. In the current context, the quickest road to prediction error minimization is to reduce the prediction error caused by on‐beat omissions (syncopations) by moving to the beat. In this way, prediction errors resulting from on‐beat syncopations are affordances, providing the opportunity to gain reward via fast and effective prediction error minimization. As such, “syncopation in groove does thus not *cause* pleasure but enables our engagement in a pleasurable activity” [78, pg. 14], that is, increasing prediction‐based learning via on‐beat movements.

## 5 Conclusion

Using a minimal Bayesian model of meter inference, we provide a process model of how meter‐based predictions drive the urge to synchronize movements to rhythmic music. This explanation supports and updates the predictive processing account of PLUMM and provides the first evidence that the urge to move is driven not by the detection of strongly weighted prediction errors, but by prediction error reduction via expected movement feedback. This aligns with the role of active inference in rhythm perception, and music listening more generally, and supports the role of learning progress in determining which musical stimuli we choose to engage with. This can be framed within a growing literature emphasizing the roles of prediction errors and uncertainty in driving musical reward [9, 10, 79, 80, 81, 82].

Ongoing and future work will test whether the model generalizes to predict other rhythmic stimuli and to account for how differences in rhythm‐related predictive processes (e.g., differences in $P_{switch}$ and C) might account for interindividual variability in the relation between rhythmic complexity and the urge to move. This work represents the first steps in establishing a comprehensive process model of PLUMM. Extensions of this model should include inference over competing meters, inference of template granularity, and processing of rhythms that are segregated into multiple auditory streams. This work can also take advantage of recent advances in modeling meter inference in continuous time, including PIPPET‐based models, which now include both tempo and phase inference [43, 56, 57]. Finally, given the importance of expected movement feedback, inference regarding the synchrony of one's own movements should be included, as this has been shown to have a strong influence on PLUMM [26].

## Author Contributions

**Tomas E. Matthews**: Writing–review and editing, writing–original draft, visualization, software, methodology, formal analysis, investigation, conceptualization. **Peter Vuust**: Funding acquisition, supervision. **Jonathan Cannon**: Writing–review and editing, supervision, methodology, visualization, software, conceptualization.

## Funding

This research was supported by the Danish National Research Foundation (DNRF 117), by the Natural Sciences and Engineering Research Council of Canada (Award Number RGPIN‐2022‐05027), and by a Human Frontier Science Program research grant (RGEC27/2025).

## Conflicts of Interest

The authors declare no competing interests.

## Supporting information




**Supplementary Figure**: nyas70129‐sup‐0001‐SuppMat.docx

## Data Availability

Code for this project can be found at https://github.com/flepid/bayesPLUMM. Data files can be found at https://osf.io/qjn6y/files/osfstorage.

## Footnotes

1 More precisely, predictive processing agents are mandated to minimize a mathematical quantity called “free energy” [83]. In continuous state‐space models, prediction error is the deviation between the continuous value of an observation and the mean of its predicted distribution, and the precision is the inverse variance of the predicted distribution; here, precision‐weighted prediction error is identical to free energy. In our discrete state‐space model, there is no neat mathematical definition of “precision‐weighted prediction error”—we use it loosely as a more intuitive surrogate for “free energy.”

2 Surprisal and precision‐weighted prediction error are not identical concepts, but they can be used interchangeably in this setting. Free energy can be expressed as a sum of two terms, where one represents the divergence between prior and posterior beliefs when sensory data is assimilated and the other represents the expected surprisal associated with that sensory data. Only the latter can be minimized through action, so for the purpose of action, the agent's mandate is to minimize expected surprisal.
