# Supplementary material for: An Active Inference Model of Meter Perception and the Urge to Move to Music
Source: Ann N Y Acad Sci. 2025 Dec 9;1556(1):e70129. doi: 10.1111/nyas.70129 (PMC12906287; doi:10.1111/nyas.70129)
Supplement: Supplementary file 1 — Supplementary Figure: nyas70129‐sup‐0001‐SuppMat.docx [file NYAS-1556-0-s001.docx]

**Supplementary Material**


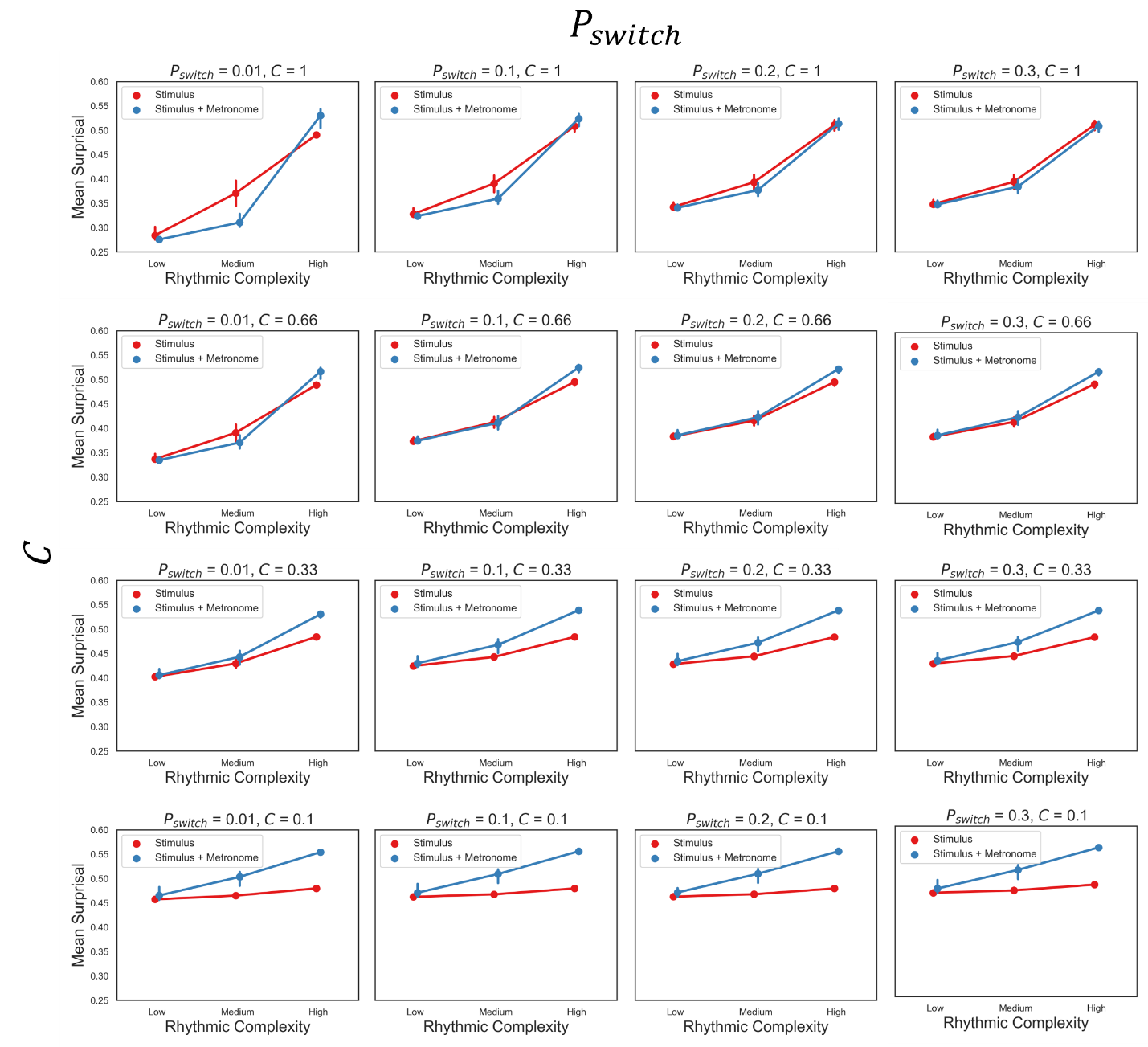


**Figure S1.** Mean surprisal plots for four levels of $P_{switch}$ (increasing across columns) and metrical contrast $C$ (decreasing across rows)**.**
